# Reactions of salicylaldehyde and enolates or their equivalents: versatile synthetic routes to chromane derivatives

**DOI:** 10.3762/bjoc.8.244

**Published:** 2012-12-12

**Authors:** Ishmael B Masesane, Zelalem Yibralign Desta

**Affiliations:** 1 Department of Chemistry, University of Botswana, Private Bag 00704, Gaborone, Botswana

**Keywords:** acetophenone, chromane, enolates, malononitrile, Michael addition, salicylaldehyde

## Abstract

The reported methodologies for the synthesis of chromane derivatives through the reaction of salicylaldehyde and enolates are discussed. The enolates and their equivalents involved in the reactions discussed in this article were derived from ketones, nitroalkanes, malononitrile and α,β-unsaturated compounds.

## Introduction

**Scheme 1:**
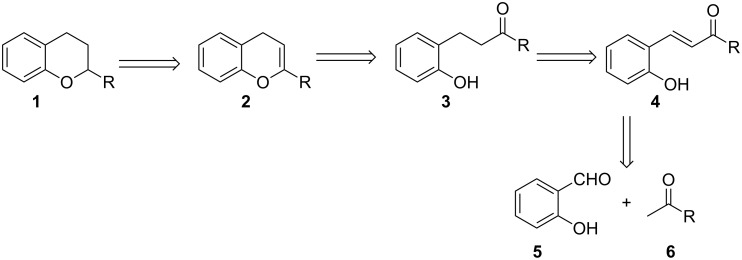
Retrosynthetic analysis of chromane **1**.

The chromane skeleton is found in a myriad of medicinally important compounds that have a broad range of biological activities [1–7]. Consequently, the synthesis of chromane derivatives has attracted the attention of synthetic chemists over the years [1–17]. Among the reported methodologies for the synthesis of chromane derivatives, the reaction of salicylaldehyde and enolates or their equivalents has gained a prominent position. The key features of the synthesis of chromane derivatives by the reaction of salicylaldehyde and enolates are summarized retrosynthetically in Scheme 1.

This review will summarize the reported methods for the syntheses of chromane derivatives from the reaction of salicylaldehyde and enolates or their equivalents. For the purposes of this review, chromane derivatives will include flavans, flavones, chromenes and chromones. The synthetic methods in the literature will be compared and contrasted in terms of their generality, selectivity and percentage yields.

## Review

### Chromane derivatives from the reaction of salicylaldehyde with enolates derived from ketones

**Scheme 2:**
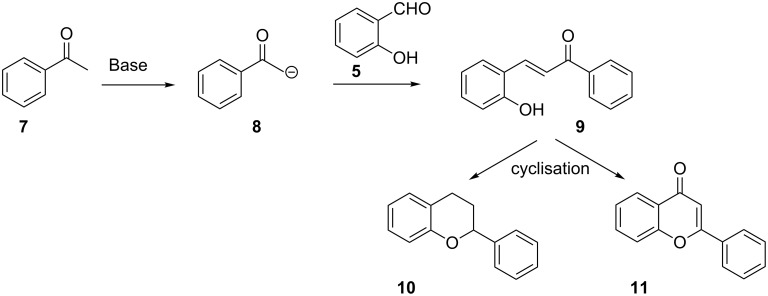
General reaction of salicylaldehyde (**5**) and acetophenone (**7**) in the synthesis of flavan **10** and flavone **11**.

The reaction of salicylaldehyde (**5**) and enolates derived from acetophenone (**7**) has been employed by a number of chemists in the synthesis of flavans and flavones. Flavans are chromane derivatives with a C-2 phenyl substituent while flavones are chromane derivatives with a carbonyl functional group at C-4, a carbon–carbon double bond between C-2 and C-3, and a C-2 phenyl substituent. The synthesis of flavans and flavones generally involves treatment of acetophenone (**7**) with a base to give enolate **8**, which undergoes a Knoevenagel condensation with salicylaldehyde (**5**) to yield a chalcone **9**. These chalcone derivatives are then cyclized by using various methodologies to give flavan **10** or flavone **11** (Scheme 2).

**Scheme 3:**
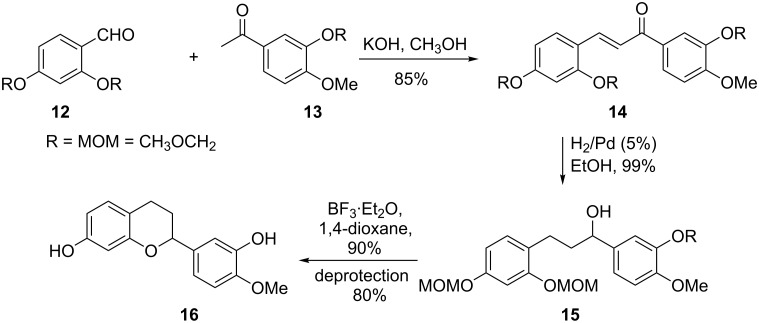
Synthesis of flavan **16** by Xue and co-workers.

Xue and co-workers have utilized the reaction of salicylaldehyde **12** and acetophenone **13** in the racemic synthesis of the naturally occurring flavan **16** (Scheme 3) [18]. To begin, a solution of **12** and **13** in CH_3_OH was stirred in the presence of KOH at room temperature to give chalcone **14**. To set the stage for the cyclisation reaction, the *trans* carbon–carbon double bond must either be isomerized to the *cis* form or completely reduced. In this case, chalcone **14** was treated with H_2_ in the presence of a catalytic amount of Pd to give intermediate **15** in 99% yield. It is instructive to draw attention to the fact that both the carbon–carbon and carbon–oxygen double bonds of **14** were reduced by H_2_/Pd, a reagent usually used for the reduction of carbon–carbon double bonds. To complete the synthesis, Lewis acid mediated cyclization of intermediate **15** and acidic cleavage of the MOM protected hydroxy group delivered the desired flavan **16** in good yield.

**Scheme 4:**
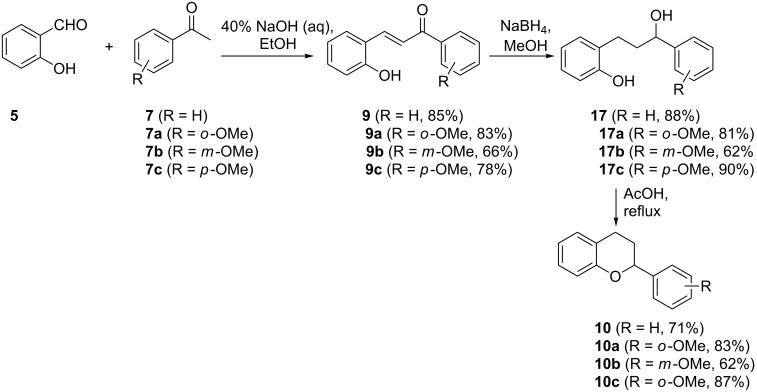
Synthesis of flavans of type **10** by Mazimba and co-workers.

On the basis of the above precedent by Xue and co-workers, our group accomplished the synthesis of an array of flavans of type **10** [19]. The synthesis begins with a Knoevenagel reaction of salicylaldehyde (**5**) and acetophenone derivatives **7** to give the corresponding chalcones of type **9** in 66–85% yields. Contrary to Xue’s reduction method where H_2_/Pd was used, we used NaBH_4_ in the reduction of both the carbon–carbon and carbon–oxygen double bonds of chalcone derivatives **9** to give the corresponding alcohols **17**. It is noteworthy that the carbon–carbon double bond was also reduced by NaBH_4_, a reagent usually used for the reduction of carbonyl groups. Cyclization was achieved by heating intermediates **17** under reflux in acetic acid to give the corresponding flavans of type **10** in 62–87% yields (Scheme 4).

**Scheme 5:**
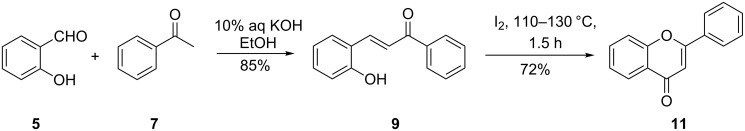
Sashidhara and co-workers synthesis of flavone (**11**).

Recently, Sashidhara and co-workers achieved the synthesis of flavone **11** relying on the reaction of salicylaldehyde (**5**) and an enolate derived from acetophenone (**7**, Scheme 5) [20]. To begin, chalcone **9** was prepared in 85% yield by the Knoevenagel reaction of salicylaldehyde (**5**) and acetophenone (**7**) in the presence of KOH (aq) in ethanol as reported by Mazimba and co-workers. Chalcone **9** was then oxidatively cyclized in the presence of iodine and in a solvent-free environment to give flavone (**11**) in 72% yield. Methyl-, methoxy- and chloro-substituted acetophenones were also well tolerated in the reaction to give the corresponding flavones in comparable yields.

**Scheme 6:**
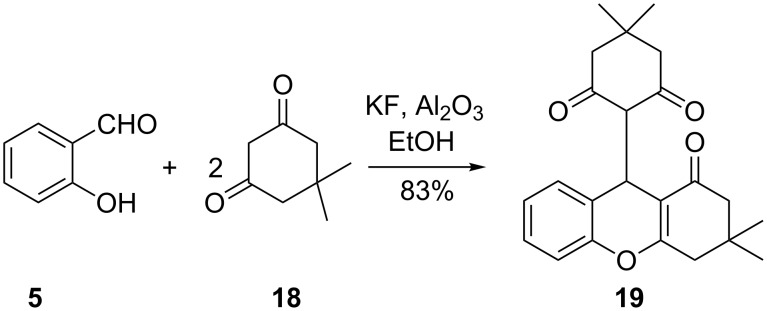
Synthesis of chromane derivative **19** by Yu-Ling and co-workers.

It is conceivable that enolates derived from other ketones instead of acetophenone could be reacted with salicylaldehyde to give chromane derivatives. To this end, Yu Ling and co-workers reported the efficient synthesis of chromane derivative **19** through the reaction of salicylaldehyde (**5**) with dimedone (**18**) in the presence of a catalytic amount of KF/Al_2_O_3_ (Scheme 6) [21]. The reaction is thought to proceed through a Knoevenagel condensation, a Michael addition and an intramolecular cyclization. The reaction was repeated with chloro-, bromo-, dichloro-, dibromo-, methyl- and nitro-substituted salicylaldehydes. The nitro- and 3,5-dibromo-substituted salicylaldehydes reacted with **18** to give the lowest yields of 60–70% while the other substituted salicylaldehydes reacted to give corresponding chromane derivatives in yields comparable to those achieved when **5** was used.

### Chromane derivatives from the reactions of salicylaldehyde and enolate equivalents derived from malononitrile and its derivatives

**Scheme 7:**
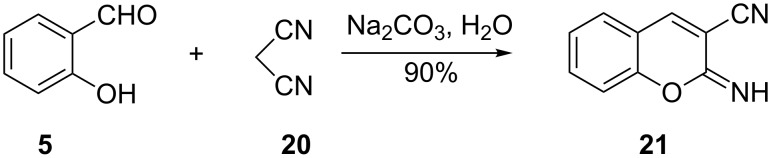
Synthesis of 2-iminochromene **21** by Costa and co-workers.

The one-pot reaction of salicylaldehyde and malononitrile has proved to be an efficient method for the synthesis of 2-iminochromene derivatives. In general such synthetic procedures involve a Knoevenagel condensation followed by intramolecular cyclization. In a detailed study directed towards understanding the pathway of the reaction of salicylaldehyde (**5**) and malononitrile (**20**), Costa and co-workers reported the efficient synthesis of 2-iminochromene **21** in 90% yield [22]. This was achieved when salicylaldehyde (**5**) was reacted with 1 equivalent of malononitrile in the presence of Na_2_CO_3_ and H_2_O as the solvent (Scheme 7). A comparable yield was obtained when NaHCO_3_ was used as the base instead of Na_2_CO_3_. The use of 3-methoxy-, 3-hydroxy-, 5-bromo-, 4-*N*,*N*-diethylamino- and 5-bromo-3-methoxy-substituted salicylaldehydes gave corresponding 2-iminochromene derivatives in 86–100% yields, while the lowest yield of 62% was achieved when 3,4-dihydroxysalicylaldehyde was used.

**Scheme 8:**
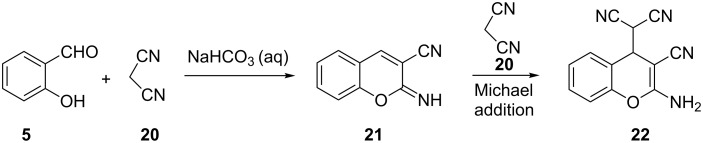
Synthesis of 2-aminochromene **22** by Costa and co-workers.

Further studies by Costa and co-workers revealed that the reaction of salicyldehyde (**5**) with 2 equivalents of malonitrile (**20**) in the presence of NaHCO_3_ afforded 2-aminochromene **22** in 91% yield (Scheme 8) [22]. This product is thought to be the result of a Michael addition of the extra malononitrile to product **21**.

**Scheme 9:**
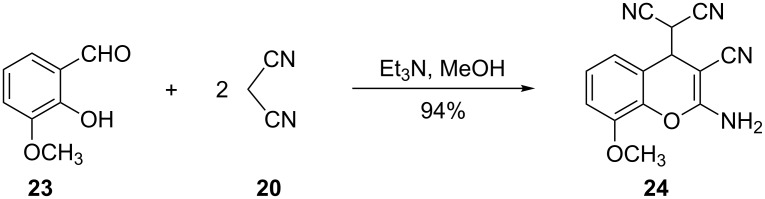
Costa and co-workers used Et_3_N in the synthesis of 2-aminochromene **24**.

In addition to inorganic bases such as Na_2_CO_3_ and NaHCO_3_, the use of amines in catalytic and quantitative amounts in the synthesis of chromane derivatives by the reaction of salicylaldehyde (**5**) and malononitrile (**20**) has been reported. Costa and co-workers used Et_3_N in the reaction of salicylaldehyde and 2 equivalents of malononitrile (**20**) in CH_3_OH to afford 2-aminochromene **24** in 94% yield (Scheme 9) [22].

**Scheme 10:**
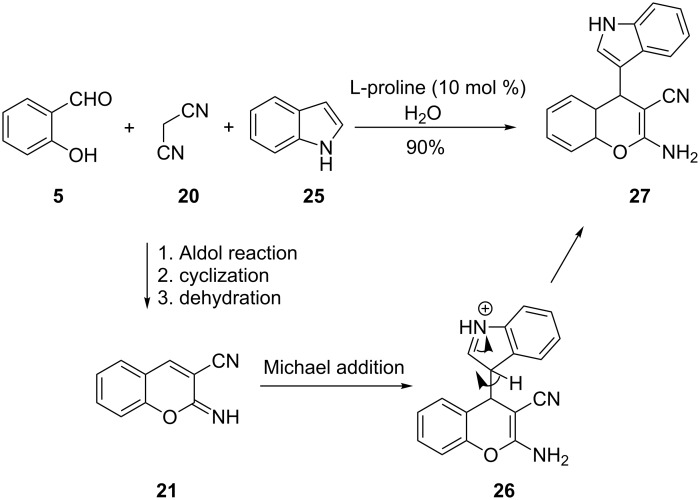
Synthesis of 2-aminochromene **27** by Shanthi and co-workers.

In 2009, Shanthi and co-workers reported the use of the amino acid L-proline as a catalyst in a three component reaction of salicylaldehyde, malononitrile and indole for the synthesis of 2-aminochromene **27** in 90% yield (Scheme 10) [23] The synthesis proceeds through a cascade reaction of salicylaldehyde (**5**) and malononitrile (**20**) involving an aldol reaction followed by intramolecular cyclization and finally a dehydration to give intermediate **21**. A subsequent Michael addition of the indole (**25**) to intermediate **21** gives cation **26**, which loses a proton to give the product **27**. Although Shanti and co-workers used a chiral catalyst, no data was provided on the stereoselectivity of this reaction.

**Scheme 11:**
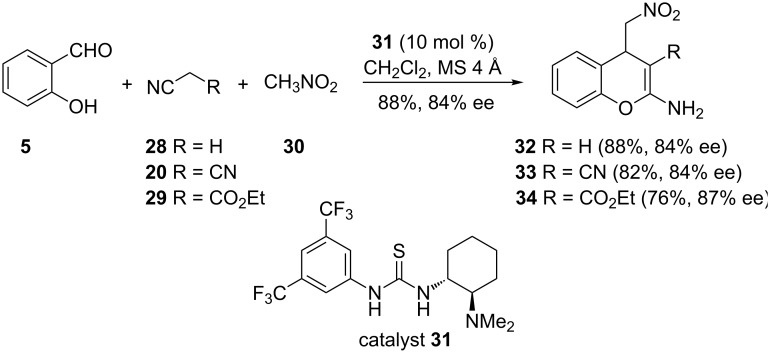
Enantioselective synthesis of 2-aminochromenes **32**–**34** by Yang and co-workers.

In a study related to that of Shanti and co-workers, Yang and co-workers used chiral amine-thiourea catalyst **31** in a three-component enantioselective reaction of salicylaldehyde (**5**), acetonitrile (**28**) and nitromethane (**30**) to give 2-aminochromene **32** in 88% yield and 84% enatiomeric excess (Scheme 11) [24]. The reaction was found to be equally efficient when malononitrile (**20**) and cyanoacetate **29** were used instead of **28**. The reaction is thought to proceed through a cascade reaction between salicylaldehyde (**5**) and acetonitrile (**28**) involving an aldol reaction, cyclization and dehydration. A subsequent Michael addition of nitromethane (**30**) to the product of the cascade reaction gave the desired product **32**.

**Scheme 12:**
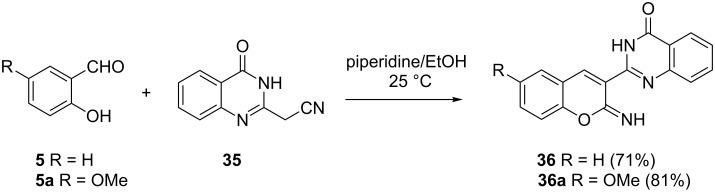
Synthesis of 2-iminochromene derivatives of type **36** by Kovalenko and co-workers.

Kovalenko and co-workers used a quantitative amount of piperidine in the reaction of malononitrile derivative **35** as an enolate equivalent and salicylaldehydes **5** to give 2-iminochromenes **36** in good yields [25]. No Michael addition product was observed. 2-hydroxy-5-methoxybenzaldehyde (**5a**) gave product **36a** in a higher yield of 81% compared to salicylaldehyde (**5**), which gave the corresponding product **36** in 71% yield (Scheme 12).

**Scheme 13:**
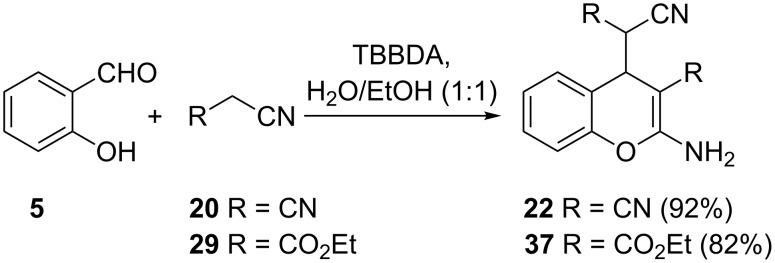
Synthesis of 2-aminochromenes **22** and **37** by Ghorbani-Vaghei and co-workers.

In another approach, Ghorbani-Vaghei and co-workers used a *N*,*N*,*N*’,*N*’-tetrabromobenzene-1,3-disulfonamide (TBBDA) mediated Knoevenagel reaction of salicylaldehyde (**5**) and two equivalents of malononitrile (**20**) or its derivative **29** to give the corresponding 2-aminochromene derivatives **22** and **37** in 92 and 82% yields respectively (Scheme 13) [26]. It is instructive to note that TBBDA is a versatile reagent in organic synthesis and has been reported to be efficient in oxidation of primary and secondary alcohols [27], in bromination of aromatic compounds [28], as catalytic reagents for silylation of alcohols, phenols, and thiols using hexamethyldisilazane [29], in conversion of urazoles to triazolinediones [30], and in oxidation of 1,3,5-trisubstituted pyrazolines [31].

**Scheme 14:**
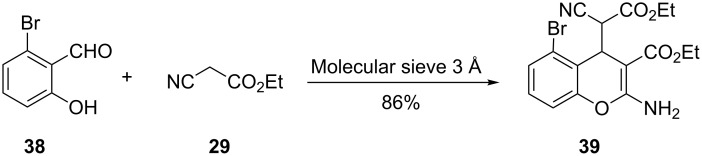
Synthesis of 2-aminochromene **39** by Yu and co-workers.

Molecular sieves have been used as solid-phase catalysts in the preparation of 2-aminochromenes from salicylaldehyde derivatives and cyanoorganic compounds. Yu and co-workers reported the one-pot synthesis of 2-aminochromene **39** in 86% yield from the reaction of bromosalicylaldehyde **38** and cyanoacetate **29** in the presence of 3 Å molecular sieves (Scheme 14) [32]. Various derivatives of **39** were prepared in good yields by employing nitro-, methoxy-, and chloro-substituted salicylaldehydes instead of **38**. Other solid catalysts such as 4 Å molecular sieves, 5 Å molecular sieves and Al_2_O_3_ were found to be effective in catalyzing the reaction but resulted in lower yields (50–63%) of product **39** [32].

**Scheme 15:**
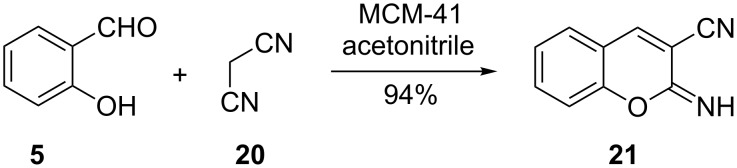
Synthesis of 2-iminochromene **21** by Heravi and co-workers.

Heravi and co-workers, on the other hand, used a mesoporous molecular sieves (MCM-41)-catalyzed Knoevenagel reaction of salicylaldehyde (**5**) and malononitrile (**20**) to give 2-iminochromene **21** in 94% yield (Scheme 15) [33]. The generality of Haravi’s method was demonstrated by the reactions of 3-hydroxy-, 4-hydroxy-, 5-hydroxy-, 4-methoxy- and 5-bromosalicylaldehyde with malononitrile (**20**) to give the corresponding 2-iminochromene derivatives in yields of at least 90%. MCM-41 can be reused for up to five cycles with an insignificant drop in percentage yields (80%).

At this juncture, it is instructive to draw attention to the fact that the yields of the molecular-sieve-catalyzed reactions of salicylaldehydes and enolate equivalents derived from malononitrile and its derivatives are comparable to those of reactions mediated by inorganic bases such as Na_2_CO_3_ (Scheme 7) and NaHCO_3_ (Scheme 8). However molecular sieves have the advantage that they are recyclable.

### Chromane derivatives from the reaction of salicylaldehyde and enolates derived from α,β-unsaturated compounds

**Scheme 16:**
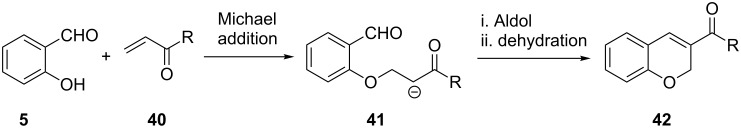
Tandem reaction of salicylaldehyde and α,β-unsaturated compounds.

The tandem reaction of salicylaldehyde and α,β-unsaturated compounds has proved to be a reliable route to chromane derivatives. In general, this reaction involves an oxo-Michael addition of salicylaldehyde (**5**) to α,β-unsaturated compounds of type **40** to give enolate intermediates of type **41**. Enolate intermediates **41** then undergo an intramolecular Knoevenagel condensation to give chromane derivatives **42** (Scheme 16).

**Scheme 17:**
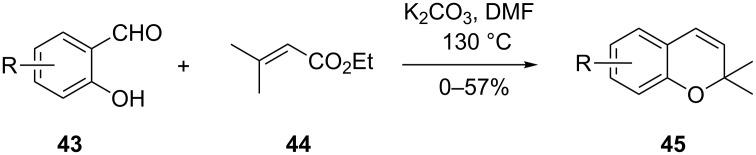
Kawase and co-workers synthesis of 2,2-dimethylchromene **45**.

Kawase and co-workers reported the K_2_CO_3_-mediated tandem reaction of salicylaldehyde derivatives of type **43** and α,β-unsaturated ester **44** in the synthesis of 2,2-dimethylchromene **45** in moderate yields (Scheme 17) [34]. The dehydration reaction in this case was accompanied by decarboxylation. The best yields were achieved when methoxy-, methyl-, chloro-, bromo- and phenyl-substituted salicylaldehydes were used as reagents. The nitro-, hydroxy-, ethoxy- and acetyl-substituted salicylaldehydes on the other hand gave poor yields or no products at all. Related reactions involving a K_2_CO_3_-mediated tandem reaction of salicylaldehyde with acrolein and alkenes with two electron withdrawing groups to give the corresponding chromane derivatives have been reported [35–37]. The percentage yields of the chromane derivatives in these reports were comparable to those reported by Kawase and co-workers.

**Scheme 18:**
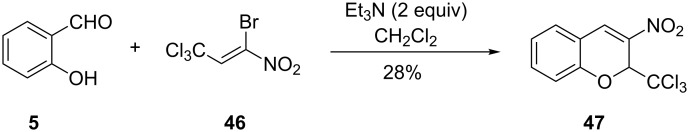
Synthesis of 2,3-disubstituted chromene **47** by Stukan and co-workers.

In addition to K_2_CO_3_, tertiary-amine-mediated tandem reactions of salicylaldehyde and α,β-unsaturated compounds to give chromane derivatives have been reported. Stukan and co-workers, for example, used an Et_3_N-mediated reaction of salicylaldehyde (**5**) and nitropropene **46** in the synthesis of 2,3-disubstituted chromene **47** in a low yield of 28% (Scheme 18) [38]. Slightly better yields (33–40%) were achieved when 5-bromo-, 5-chloro- and 3,5-dichloro-substituted salicylaldehydes were employed in the reaction.

**Scheme 19:**
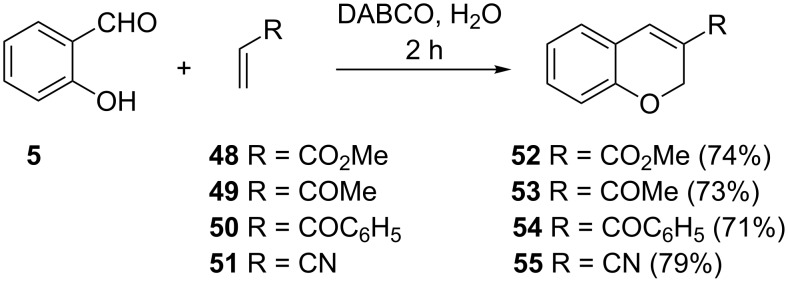
Ravichandrans synthesis of 3-substituted chromenes **52–55**.

Ravichandran utilized a classical 1,4-diazabicyclo[2.2.2]octane (DABCO)-catalyzed Baylis–Hillman reaction of salicylaldehyde (**5**) and α,β-unsaturated compounds **48–51** in the synthesis of the corresponding chromenes **52–55** (Scheme 19) [39]. These reactions were performed in water as the solvent and the chromenes were isolated in yields of 71–79%. It is instructive to note that the Baylis–Hillman products were not detected or isolated in this work.

**Scheme 20:**
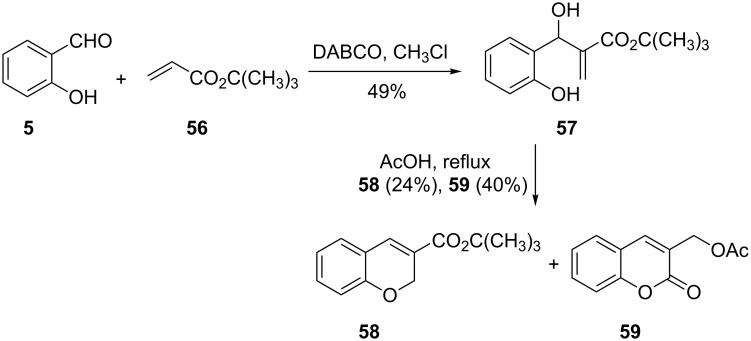
Synthesis of 3-substituted chromene **58** coumarin **59** by Paye and co-workers.

The mechanism of the DABCO-catalyzed reaction of salicylaldehyde and α,β-unsaturated compounds in the synthesis 3-substituted chromenes was proved to proceed through the Baylis–Hillman reaction by Kaye and co-workers [40–41] Their work involved the reaction of salicylaldehyde (**5**) with *tert*-butyl acrylate (**56**) to give the Baylis–Hillman product **57**, which was subsequently cyclized in the presence of acetic acid to give chromene **58** in a low yield of 24%, together with coumarin **59** in 40% yield (Scheme 20).

**Scheme 21:**
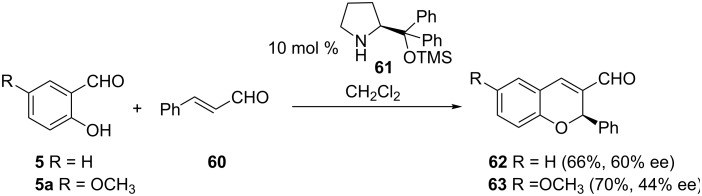
Govender and co-workers asymmetric synthesis of 2-phenylchromenes **62** and **63**.

An asymmetric amine-catalyzed reaction of salicylaldehyde (**5**) and α,β-unsaturated aldehyde **60** in the synthesis of 2-phenylchromene (**62**) was reported by Govender and co-workers [42]. The asymmetric union of salicylaldehyde (**5**) and aldehyde **60** was brought about by dissolving these two substances in CH_2_Cl_2_ in the presence of catalytic amounts of TMS-protected prolinol derivative **61** (Scheme 21). Methoxysalicylaldehyde **5a** reacted much faster than salicylaldehyde (**5**) with higher isolated yield of 2-phenychromene **63** but at the expense of enantioselectivity. The best enantioselectivity (90% ee) was achieved when the aliphatic aldehyde 2-hexenal was used in the reaction instead of **60**. However, the reaction suffered from very poor yields (15–21%). The reaction is thought to proceed through the condensation of aldehyde **60** and prolinol **61** to give a chiral iminium-ion intermediate. This intermediate then undergoes a domino reaction involving a Michael reaction with salicylaldehyde (**5**), followed by an intramolecular aldol reaction and final dehydration to give the desired chromene derivative.

**Scheme 22:**
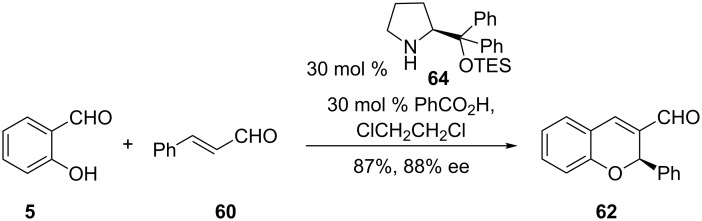
Asymmetric synthesis of 2-phenylchromene **62** by Li and co-workers.

Related work involving asymmetric reaction of salicylaldehyde derivatives and α,β-unsaturated carbonyl compounds in the synthesis of 2-phenylchromenes was reported by Li and co-workers (Scheme 22) [43]. Their strategy involved the reaction of salicylaldehyde (**5**) and unsaturated aldehyde **60** in the presence of catalytic amounts of TES-protected prolinol **64** and benzoic acid. High yields (87%) and excellent enantioselectivity (88%) of 2-phenylchromene **62** were achieved when the reaction was performed in 1,2-dichloroethane as the solvent. The presence of the benzoic acid additive is thought to be responsible for the increase in the enantioselectivity and higher yields of this reaction when compared to that of Govender and co-workers. It is also instructive to note that the catalyst loading for Li and co-workers was three times higher than that for Govender and co-workers.

## Conclusion

This paper has demonstrated the versatility of the reactions of salicylaldehyde with enolates or their equivalents in the synthesis of chromane derivatives. These reactions can be run under quite mild conditions and are ideal for the synthesis of chromane derivatives due to their operational simplicity. The development of enantioselective reactions of salicylaldehyde and enolates to give nearly optically pure chromane derivatives is a memorable highlight of this review. Future work will undoubtedly focus on transformation of the products of the discussed reactions of salicylaldehyde with enolates to biologically active compounds and natural products.
